# *In vitro* efficacy of relebactam versus avibactam against *Mycobacterium abscessus* complex

**DOI:** 10.1016/j.tcsw.2021.100064

**Published:** 2021-10-09

**Authors:** James Harrison, John A. Weaver, Maya Desai, Jonathan A.G. Cox

**Affiliations:** a College of Health and Life Sciences, Aston University, Aston Triangle, Birmingham B4 7ET, UK; b Birmingham Children’s Hospital, Birmingham Women’s and Children’s NHS Foundation Trust, Steelhouse Lane, Birmingham B4 6NH, UK

## Abstract

Infections resulting from *Mycobacterium abscessus* are increasing in prevalence worldwide, with the greatest risk posed to patients with underlying respiratory conditions. Treatment for infections is difficult due to wide ranging intrinsic antimicrobial resistance, which is compounded by the existence of a range of subspecies within the *M. abscessus* complex, each with varying additional antimicrobial resistance profiles. Previously, the use of β-lactam/β-lactamase inhibitors within a combination therapy has been proposed as an effective treatment option for pulmonary *M. abscessus* infections. Here, we assess the *in vitro* efficacy of two non-β-lactam based inhibitors, relebactam and avibactam, as agents against *M. abscessus* with their respective partner drugs imipenem and ceftazidime, as well as in triplicate combinations with additional β-lactam antibiotics against the *M. abscessus* complex. We have shown that the commercially available ratio of imipenem to relebactam is the appropriate ratio for bactericidal activity against *M. abscessus*, whereas the ratio between ceftazidime and avibactam is redundant, due to inactivity of ceftazidime to inhibit the bacteria. We have identified that the use of imipenem and meropenem alongside either relebactam or avibactam yield low minimum inhibitory concentrations (MIC) and minimum bactericidal concentrations (MBC) for each *M. abscessus* subspecies, which are within the therapeutically achievable concentration ranges within the epithelial lining fluid of the lungs. We propose the implementation of imipenem with relebactam in place of stand-alone imipenem into the current treatment regime, alongside meropenem, as a future front-line treatment option for *M. abscessus* complex infections.

## 1 Introduction

*Mycobacterium abscessus* is an opportunistic human pathogen that is an increasing global health threat, capable of causing pulmonary disease or skin and soft tissue infections. Of particular susceptibility to these infections are those who suffer from cystic fibrosis (CF) or bronchiectasis (Viviani et al., 2016, Gardner et al., 2019). High morbidity due to *M. abscessus* infection can be attributed to the difficulty in treatment with antimicrobials, largely in part due to the multiple antibiotic resistance mechanisms employed by the pathogen (Lopeman et al., 2019). The prolonged treatment regime consists of a combination therapy of amikacin, tigecycline and imipenem, as well as clarithromycin or azithromycin (if susceptible) for one month. This is then followed up with a combination of clofazimine, linezolid, minocycline, moxifloxacin or co-trimoxaole alongside nebulised amikacin, (dependent upon isolate susceptibility profiling) for 12-months (Haworth et al., 2017, Chen et al., 2019). The difficulty in isolate susceptibilities is exacerbated by the existence of differing subspecies of *M. abscessus*, which have subtle genetic variations giving rise to phenotypic differences within the population. *M. abscessus* subsp. *abscessus* is the most common variant, whereas *M. abscessus* subsp. *bolletii* is the rarest (Adékambi et al., 2006, Tortoli et al., 2016, Minias et al., 2020). Both of these subspecies contain a functional *erm(41)* gene encoding for inducible macrolide resistance. However, *M. abscessus* subsp. *massiliense* does not have a functional *erm(41)* gene, and as such, possesses no resistance to macrolide antibiotics (Nash et al., 2009, Luthra et al., 2018, Bordin et al., 2021). It is therefore an important consideration to identify subspecies susceptibilities to not only the front line treatment options, but also any drug discovery efforts to combat *M. abscessus*.

A major mechanism of antimicrobial resistance employed by *M. abscessus* is the intrinsic β-lactam resistance provided by expression of an endogenous class A β-lactamase, Bla_Mab_, which hydrolyses common antibiotics such as penicillin and renders them ineffective (Soroka et al., 2014). Since the discovery of Bla_Mab_ there has been an effort to exploit the inhibition of the protein for treatment options (Soroka et al., 2014, Dubée et al., 2015). The most promising of these β-lactamase inhibitors to date are the non-β-lactam based inhibitors, such as avibactam, relebactam and vaborbactam (Wang et al., 2016, Wong and van Duin, 2017). These β-lactamase inhibitors have been shown to be effective at increasing the susceptibility of *M. abscessus* to multiple β-lactam antibiotics, in particular carbapenems such as meropenem and imipenem (Dubée et al., 2015, Kaushik et al., 2019).

Avibactam has been demonstrated to be capable of reducing the required concentrations of carbapenems down to therapeutically relevant levels in *M. abscessus*, in particular increasing the susceptibility to panipenem, ertapenem and tebipenem (Kaushik et al., 2017). The efficacy of both imipenem and amicakin when used together against *M. abscessus* was also shown to be improved when used in conjunction with avibactam (Lefebvre et al., 2017). Previously, we have shown that the use of relebactam also increases the susceptibility of *M. abscessus* to amoxicillin, which does not have any effect on the bacteria in the absence of the β-lactamase inhibitor. Importantly, this was also seen across a panel of clinical *M. abscessus* isolates (Lopeman et al., 2020).

In this study, we sought to identify the most potent β-lactamase inhibitor combination against the three subspecies of the *M. abscessus* complex, including either relebactam or avibactam. We aim to establish the most effective ratios of β-lactam to β-lactamase inhibitor of the commercially available formulations for both of these compounds against *M. abscessus*. This research also explores the relationship between these β-lactamase inhibitors with dual β-lactam partners against clinically relevant *M. abscessus* subspecies, in order to assess the efficacy of these triplicate combinations. Our aim for this *in vitro* assessment is to highlight the chemotherapeutic potential of β-lactamase inhibitor combinations for treatment of *M. abscessus* infections and to provide an evidence base for the selection of β-lactamase inhibitor combinations.

## 2 Materials and methods

### 2.1 Bacterial isolates

*M. abscessus* NCTC 13031, 15944 subsp. *abscessus*, DC088A subsp. *bolletii*, DC088D subsp. *massiliense* were routinely grown in Middlebrook 7H9 containing 10 % (v/v) Albumin-Dextrose-Catalase (ADC) supplement, 1 % (w/v) glycerol and 0.05 % (w/v) Tween80 and incubated at 37 °C with orbital shaking at 180 rpm.

### 2.2 Antimicrobials

The antimicrobials ceftazidime (CAZ) and amoxicillin (AMX) were sourced from Sigma Aldrich (Dorset, UK). Imipenem (IMI), meropenem (MER), avibactam (AVI) and relebactam (REL) were obtained from Carbosynth (Compton, UK). Stock solutions of all compounds were prepared in sterile dH_2_O, apart from AMX where DMSO was used, and stored at −20 °C, where appropriate.

### 2.3 Broth microdilution assays

Broth microdilution assays (or checkerboard assays) were performed as previously described, with suitable modifications for this study (Lopeman et al., 2020). Briefly, 96-well plates were prepared by serial dilution of MER (32, 16, 8, 4, 2, 1, 0.5, 0 µg/mL), AMX (128, 64, 32, 16, 8, 4, 2, 0 µg/mL), CAZ (24, 12, 6, 3, 1.5, 0.75, 0.375, 0 µg/mL) or IMI (24, 12, 6, 3, 1.5, 0.75, 0.375, 0 µg/mL), along the x-axis and either CAZ/AVI (48/12, 24/6, 12/3, 6/1.5, 3/0.75, 1.5/0.375, 0.75/0.1875, 0/0 µg/mL), IMI/REL (24/12, 12/6, 6/3, 3/1.5, 1.5/0.75, 0.75/0.375, 0.375/0.1875, 0/0 µg/mL), IMI/AVI (24/12, 12/6, 6/3, 3/1.5, 1.5/0.75, 0.75/0.375, 0.375/0.1875, 0/0 µg/mL), AVI (24, 12, 6, 3, 1.5, 0.75, 0.375, 0 µg/mL), or REL (24, 12, 6, 3, 1.5, 0.75, 0.375, 0 µg/mL), along the y-axis, depending upon the compound combination being analysed. *M. abscessus* isolates were diluted to OD_600 nm_ = 0.1 before addition to experimental wells at a final volume of 100 µL (n = 4). Plates were sealed and incubated at 37 °C for 96 h. Spectrophotometric plate reads at 570 nm were taken every 24 h. Experimental wells at the minimum inhibitory concentration (MIC) were retroactively plotted as absorbance vs time using GraphPad Prism 8. The broth microdilution assay was repeated for each *M. abscessus* subspecies (n = 4).

### 2.4 Minimum bactericidal concentration evaluation

The Minimum Bactericidal Concentration (MBC) of each combination was assessed by 5 µL of each experimental well being spotted onto Middlebrook 7H11 agar plates after 96 h growth. The spots were dried before incubation of the agar plates at 37 °C for 48 h. MBC values were determined as the lowest concentrations with an absence of bacterial growth.

## 3 Results

### 3.1 Assessment of β-lactam/β-lactamase inhibitor formulations against *M. Abscessus*

**Fig. 1:**
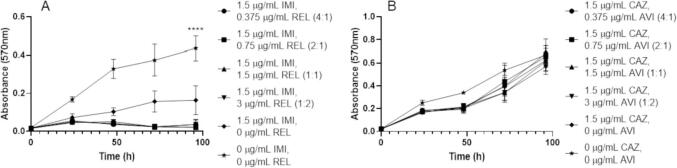
Growth curves of *M. abscessus* NCTC 13031 with varying β-lactam/β-lactamase inhibitor combination ratios. A) Varying ratios of imipenem (IMI) and relebactam (REL) (p ≤ 0.0001, n = 4). B) Varying ratios of ceftazidime (CAZ) and avibactam (AVI) (p = 0.5287, n = 4).

The β-lactam/β-lactamase inhibitor combinations of IMI/REL and CAZ/AVI are pre-formulated at 2:1 and 4:1 ratios respectively (Vazquez et al., 2012, Lucasti et al., 2016). However, since these formulations were created to treat infections other than those caused by *M. abscessus*, we investigated the role of differing β-lactam/β-lactamase inhibitor ratios against this organism *in vitro*. Using microbroth dilution checkerboard assays, we assessed different concentrations of IMI and REL as well as CAZ and AVI at ratios other than those in which they are pre-formulated as. IMI has potent activity against *M. abscessus* NCTC with small concentrations of REL, with activity as low as a ratio of 4:1 (IMI:REL) (Fig. 1). This inhibitory activity is beyond that of IMI alone and is comparable to the activity of ratios of 2:1 (available formulation), 1:1 and 1:2 (IMI:REL) (Fig. 1). However, the minimum concentrations at which bactericidal activity was observed were 3 µg/mL IMI, 1.5 µg/mL REL (2:1 (IMI:REL) ratio), whereas higher concentrations of REL were not able to reduce the minimum required concentration of IMI needed to sterilise *M. abscessus* and lower REL concentrations with 3 µg/mL IMI did not have bactericidal activity (Table 1). Ratios of CAZ and AVI did not have any inhibitory activity against *M. abscessus* NCTC as high as 4:1 (combination pre-formulation) (Fig. 1). None of these concentrations were bactericidal against *M. abscessus* (Table 1).

**Table 1:** Minimum inhibitory concentrations (MIC) and minimum bactericidal concentrations (MBC) of each component of assessed drug combinations against *M. abscessus* complex.

*M. abscessus* subspecies								
Drug (combo)	NCTC		subsp. *abscessus*		subsp. *bolletii*		subsp. *massiliense*	
	MIC (µg/mL)	MBC (µg/mL)	MIC (µg/mL)	MBC (µg/mL)	MIC (µg/mL)	MBC (µg/mL)	MIC (µg/mL)	MBC (µg/mL)
CAZ	>48	>48	>48	>48	>48	>48	>48	>48
IMI	3	3	1.5	3	3	3	1.5	6
MER	6	6	6	6	12	24	12	12
AMX	>128	>128	>128	>128	>128	>128	>128	>128
CAZ/AVI	>48/>24	>48/>24	>48/>24	>48/>24	>48/>24	>48/>24	>48/>24	>48/>24
IMI/MER	1.5/1.5	1.5/1.5	0.375/1.5	1.5/0.75	1.5/3	1.5/0.75	0.75/1.5	1.5/3
CAZ/AVI (MER)	0.75/0.1875	0.75/0.1875	0.75/0.1875	0.75/0.1875	0.75/0.1875	0.75/0.1875	0.75/0.1875	0.75/0.1875
CAZ/AVI (IMI)	1.5/0.375	0.75/0.1875	0.75/0.1875	0.75/0.1875	0.75/0.1875	0.75/0.1875	0.75/0.1875	0.75/0.1875
CAZ/AVI (AMX)	3/0.75	3/0.75	1.5/0.375	1.5/0.375	6/1.5	6/1.5	3/0.75	3/0.75
MER (CAZ/AVI)	2	2	2	2	2	2	4	4
IMI (CAZ/AVI)	1.5	3	1.5	3	1.5	3	1.5	6
AMX (CAZ/AVI)	8	8	16	16	16	16	16	16
IMI/REL	0.375	3/1.5	0.375	6/0.375	1.5/0.375	6/0.375	1.5/1.5	12/0.75
IMI/REL (MER)	0.75/0.375	0.75/0.375	0.75/0.375	0.75/0.375	0.75/0.375	0.75/0.375	0.75/0.375	0.75/0.375
IMI/REL (AMX)	1.5/0.75	1.5/0.75	1.5/0.75	1.5/0.75	1.5/0.75	3/1.5	3/1.5	6/3
MER (IMI/REL)	1	1	1	1	1	1	1	1
AMX (IMI/REL)	8	16	16	64	64	128	16	0
IMI/AVI	3/1.5	0.75/0.375	3/1.5	0.75/0.375	6/3	0.75/0.375	3/1.5	1.5/0.75
IMI/AVI (MER)	0.375/0.1875	0.375/0.1875	0.375/0.1875	0.375/0.1875	0.375/0.1875	0.375/0.1875	0.75/0.375	0.75/0.375
IMI/AVI (AMX)	1.5/0.75	1.5/0.75	0.75/0.375	1.5/0.75	1.5/0.75	1.5/0.75	1.5/0.75	1.5/0.75
MER (IMI/AVI)	0.5	0.5	0.5	0.5	0.5	0.5	0.5	0.5
AMX (IMI/AVI)	8	8	16	16	16	32	32	32

### 3.2 Combinations with meropenem

**Fig. 2:**
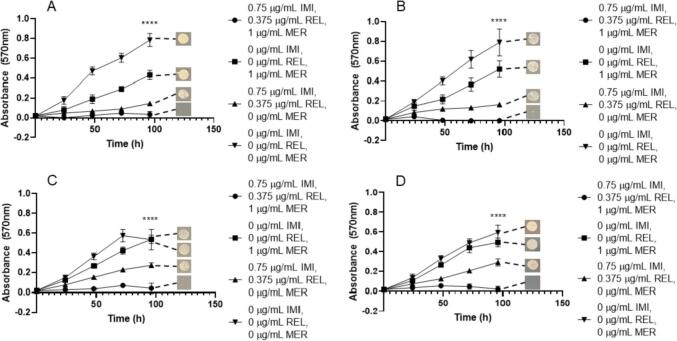
Growth curves of *M. abscessus* subspecies with imipenem (IMI)/relebactam (REL) vs meropenem (MER). Endpoint solid media bacterial re-growth is shown at the end of each curve. A) *M. abscessus* NCTC 13031 with an MIC of 0.75 µg/mL IMI, 0.375 µg/mL REL and 1 µg/mL MER (p ≤ 0.0001, n = 4). B) *M. abscessus* subsp. *abscessus* with an MIC of 0.75 µg/mL IMI, 0.375 µg/mL REL and 1 µg/mL MER (p ≤ 0.0001, n = 4). C) *M. abscessus* subsp. *bolletii* with an MIC of 0.75 µg/mL IMI, 0.375 µg/mL REL and 1 µg/mL MER (p ≤ 0.0001, n = 4). D) *M. abscessus* subsp. *massiliense* with an MIC of 0.75 µg/mL IMI, 0.375 µg/mL REL and 1 µg/mL MER (p ≤ 0.0001, n = 4).

Using the appropriate pre-formulation ratios of β-lactam to β-lactamase inhibitor partnerships, to provide the most clinically relevant data, we aimed to investigate whether these were being complemented by the appropriate antimicrobial compound in their respective proposed triplicate combinations. We assessed both the combination of CAZ/AVI with MER, as well as IMI/REL with MER against each subspecies of *M. abscessus* in order to evaluate the combination that could provide the best clinical application. The lowest MIC of MER at 1 µg/mL was achieved by combination with IMI/REL at 0.75 µg/mL/0.375 µg/mL respectively for every subspecies; *M. abscessus* NCTC, *M. abscessus* subsp. *abscessus, M. abscessus* subsp. *bolletii* and *M. abscessus* subsp. *massiliense* (Fig. 2). The concentrations of IMI (0.75 µg/mL) and REL (0.375 µg/mL) without MER were not sufficient to completely inhibit growth of any of *M. abscessus* subsp., as was also the case for MER only (1 µg/mL) (Fig. 2)*.* These combination concentrations are bactericidal, yielding combined minimum bactericidal concentrations (MBCs) of 0.75 µg/mL IMI, 0.375 µg/mL REL and 1 µg/mL MER in each subspecies of *M. abscessus* (Table 1).

**Fig. 3:**
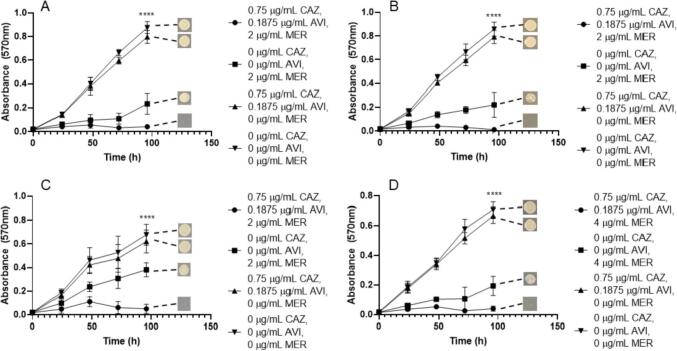
Growth curves of *M. abscessus* subspecies with ceftazidime (CAZ)/avibactam (AVI) vs meropenem (MER). Endpoint solid media bacterial re-growth is shown at the end of each curve. A) *M. abscessus* NCTC 13031 with an MIC of 0.75 µg/mL CAZ, 0.1875 µg/mL AVI and 2 µg/mL MER (p ≤ 0.0001, n = 4). B) *M. abscessus* subsp. *abscessus* with an MIC of 0.75 µg/mL CAZ, 0.1875 µg/mL AVI and 2 µg/mL MER (p ≤ 0.0001, n = 4). C) *M. abscessus* subsp. *bolletii* with an MIC of 0.75 µg/mL CAZ, 0.1875 µg/mL AVI and 2 µg/mL MER (p ≤ 0.0001, n = 4). D) *M. abscessus* subsp. *massiliense* with an MIC of 0.75 µg/mL CAZ, 0.1875 µg/mL AVI and 4 µg/mL MER (p ≤ 0.0001, n = 4).

In conjunction with CAZ/AVI, the lowest MIC of MER was 2 µg/mL at 0.75 µg/mL and 0.1875 µg/mL for CAZ and AVI respectively. These concentrations were required for the inhibition of *M. abscessus* NCTC, *M. abscessus* subsp. *abscessus* and *M. abscessus* subsp. *bolletii* (Fig. 3). However, slightly higher concentration of MER (4 µg/mL) was required to inhibit growth of *M. abscessus* subsp. *massiliense* at the equivalent concentrations of CAZ/AVI as the other subspecies (Fig. 3). In all subspecies, MER (at 2 µg/mL and 4 µg/mL) in the absence of CAZ and AVI resulted in some inhibition of bacterial growth, but not to the extent of the triplicate combination, whereas CAZ/AVI in the absence of MER resulted in no reduction in growth (Fig. 3). Concentrations of 0.75 µg/mL CAZ, 0.1875 µg/mL AVI and 2 µg/mL MER were bactericidal against each *M. abscessus* subspecies, apart from *M. abscessus* subsp. *massiliense* which required 4 µg/mL MER at the equivalent concentrations of CAZ and AVI (0.75 µg/mL and 0.1875 µg/mL respectively) (Table 1).

**Fig. 4:**
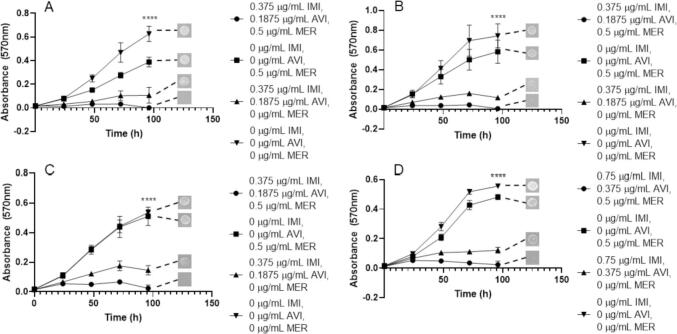
Growth curves of *M. abscessus* subspecies with imipenem (IMI)/avibactam (AVI) vs meropenem (MER). Endpoint solid media bacterial re-growth is shown at the end of each curve. A) *M. abscessus* NCTC 13031 with an MIC of 0.375 µg/mL IMI, 0.1875 µg/mL AVI and 0.5 µg/mL MER (p ≤ 0.0001, n = 4). B) *M. abscessus* subsp. *abscessus* with an MIC of 0.375 µg/mL IMI, 0.1875 µg/mL AVI and 0.5 µg/mL MER (p ≤ 0.0001, n = 4). C) *M. abscessus* subsp. *bolletii* with an MIC of 0.375 µg/mL IMI, 0.1875 µg/mL AVI and 0.5 µg/mL MER (p ≤ 0.0001, n = 4). D) *M. abscessus* subsp. *massiliense* with an MIC of 0.75 µg/mL IMI, 0.1875 µg/mL AVI and 0.5 µg/mL MER (p ≤ 0.0001, n = 4).

Given the efficacy of MER as a companion β-lactam with both IMI/REL and CAZ/AVI, an assessment of AVI with IMI (1:2 respectively) was made versus MER. MICs of 0.375 µg/mL for IMI, 0.1875 µg/mL for AVI and 0.5 µg/mL for MER were recorded for *M. abscessus* NCTC, *M. abscessus* subsp. *abscessus* and *M. abscessus* subsp. *bolletii* (Fig. 4)*.* Slightly higher concentrations of both IMI (0.75 µg/mL) and AVI (0.375 µg/mL) were required against *M. abscessus* subsp. *massiliense* (Fig. 4). IMI with AVI at 0.375 µg/mL and 0.1875 µg/mL, respectively, were able to inhibit the growth of all *M. abscessus* subspecies, but not to the extent as when combined with MER (Fig. 4). However, MER alone at 0.5 µg/mL was not sufficient to greatly inhibit the growth of any of *M. abscessus* (Fig. 4). These concentrations were bactericidal against each corresponding *M. abscessus* subspecies (Table 1).

### 3.3 Amoxicillin combinations

**Fig. 5:**
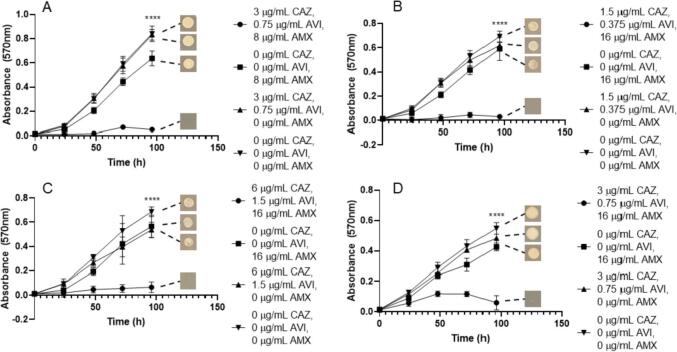
Growth curves of *M. abscessus* subspecies with ceftazidime (CAZ)/avibactam (AVI) vs amoxicillin (AMX). Endpoint solid media bacterial re-growth is shown at the end of each curve. A) *M. abscessus* NCTC 13031 with an MIC of 3 µg/mL CAZ, 0.75 µg/mL AVI and 8 µg/mL AMX (p ≤ 0.0001, n = 4). B) *M. abscessus* subsp. *abscessus* with an MIC of 1.5 µg/mL CAZ, 0.375 µg/mL AVI and 16 µg/mL AMX (p ≤ 0.0001, n = 4). C) *M. abscessus* subsp. *bolletii* with an MIC of 6 µg/mL CAZ, 1.5 µg/mL AVI and 16 µg/mL AMX (p ≤ <0.0001, n = 4). D) *M. abscessus* subsp. *massiliense* with an MIC of 3 µg/mL CAZ, 0.75 µg/mL AVI and 16 µg/mL AMX (p ≤ 0.0001, n = 4).

Since these β-lactamase inhibitors have been shown to increase the susceptibility of *M. abscessus* to AMX, we then investigated the efficacies of both CAZ/AVI with AMX and IMI/REL with AMX against the *M. abscessus* subspecies. We identified that the MIC for *M. abscessus* NCTC required 3 µg/mL CAZ, 0.75 µg/mL AVI, along with 8 µg/mL AMX, which was not the same for *M. abscessus* subsp. *abscessus*, which required 1.5 µg/mL CAZ, 0.375 AVI and 16 µg/mL AMX (Fig. 5). *M. abscessus* subsp. *massiliense* required slightly more AMX than *M. abscessus* NCTC (16 µg/mL), but the same concentrations of CAZ and AVI (Fig. 5). However, *M. abscessus* subsp. *bolletii* required higher concentrations than the other subspecies for each combination compound, with an MIC of 6 µg/mL CAZ, 1.5 µg/mL AVI with 16 µg/mL AMX (Fig. 5). Neither CAZ/AVI alone (up to 6 µg/mL and 1.5 µg/mL) nor AMX alone (up to 16 µg/mL) were capable of inhibiting bacterial growth (Fig. 5). These combined MIC concentrations were all bactericidal in each corresponding *M. abscessus* subspecies (Table 1).

**Fig. 6:**
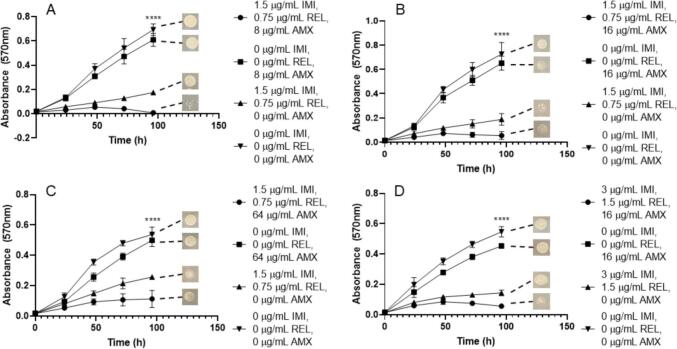
Growth curves of *M. abscessus* subspecies with imipenem (IMI)/relebactam (REL) vs amoxicillin (AMX). Endpoint solid media bacterial re-growth is shown at the end of each curve. A) *M. abscessus* NCTC 13031 with an MIC of 1.5 µg/mL IMI, 0.75 µg/mL REL and 8 µg/mL AMX (p ≤ 0.0001, n = 4). B) *M. abscessus* subsp. *abscessus* with an MIC of 1.5 µg/mL IMI, 0.75 µg/mL REL and 16 µg/mL AMX (p ≤ 0.0001, n = 4). C) *M. abscessus* subsp. *bolletii* with an MIC of 1.5 µg/mL IMI, 0.75 µg/mL REL and 64 µg/mL AMX (p ≤ 0.0001, n = 4). D) *M. abscessus* subsp. *massiliense* with an MIC of 3 µg/mL IMI, 1.5 µg/mL REL and 16 µg/mL AMX (p ≤ 0.0001, n = 4).

This trend is similarly seen when assessing the combination of IMI/REL with AMX against each of the *M. abscessus* subspecies. The concentrations of IMI/REL required at the MIC for *M. abscessus* NCTC are much lower than that of CAZ/AVI (1.5 µg/mL and 0.75 µg/mL respectively), however the same concentration of AMX is needed (8 µg/mL) (Fig. 6). The MIC of AMX required then increased for the remaining subspecies, 16 µg/mL for *M. abscessus* subsp. *abscessus* and 64 µg/mL for *M. abscessus* subsp. *bolletii*, with the same concentrations of IMI/REL. However, *M. abscessus* subsp. *massiliense* required an increase in IMI/REL concentration to 3 µg/mL and 1.5 µg/mL respectively, alongside 16 µg/mL AMX (Fig. 6). IMI and REL alone (at concentrations of both 1.5 µg/mL/0.75 µg/mL (IMI/REL) and 3 µg/mL/1.5 µg/mL) were able to inhibit the growth of all *M. abscessus* subspecies, however not to the extent of the triplicate combination (Fig. 6). Conversely, AMX alone (up to 64 µg/mL) was not sufficient to inhibit the growth of *M. abscessus* (Fig. 6). The concentrations of IMI/REL and AMX required to become bactericidal are increased from the MIC values. Concentrations of 1.5 µg/mL IMI, 0.75 µg/mL REL and 16 µg/mL AMX are required to be lethal to *M. abscessus* NCTC, whereas 64 µg/mL is needed against *M. abscessus* subsp. *abscessus*. An increase of all concentrations are required to be bactericidal against *M. abscessus* subsp. *bolletii*, to 3 µg/mL IMI, 1.5 µg/mL REL and 128 µg/mL AMX (Table 1). Interestingly, there is no benefit to the inclusion of AMX in the combination to be bactericidal against *M. abscessus* subsp. *massiliense*, since the MBC is 6 µg/mL IMI, 3 µg/mL REL and 0 µg/mL AMX (Table 1).

**Fig. 7:**
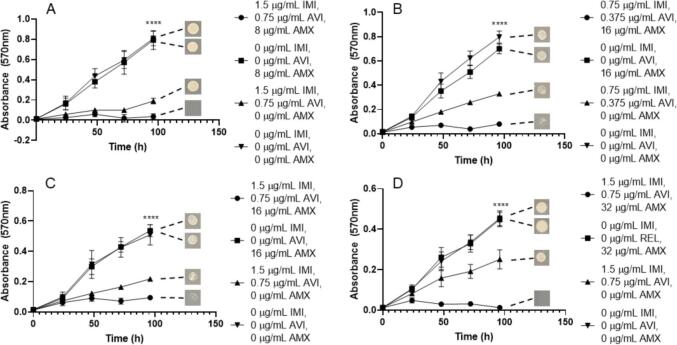
Growth curves of *M. abscessus* subspecies with imipenem (IMI)/avibactam (AVI) vs amoxicillin (AMX). Endpoint solid media bacterial re-growth is shown at the end of each curve. A) *M. abscessus* NCTC 13031 with an MIC of 1.5 µg/mL IMI, 0.75 µg/mL AVI and 8 µg/mL AMX (p ≤ =<0.0001, n = 4). B) *M. abscessus* subsp. *abscessus* with an MIC of 0.75 µg/mL IMI, 0.375 µg/mL AVI and 16 µg/mL AMX (p ≤ 0.0001, n = 4). C) *M. abscessus* subsp. *bolletii* with an MIC of 1.5 µg/mL IMI, 0.75 µg/mL AVI and 16 µg/mL AMX (p ≤ 0.0001, n = 4). D) *M. abscessus* subsp. *massiliense* with an MIC of 1.5 µg/mL IMI, 0.75 µg/mL AVI and 32 µg/mL AMX (p ≤ 0.0001, n = 4).

We also investigated the efficacy of IMI in partnership with AVI (2:1) versus AMX against the *M. abscessus* subspecies. MIC values were largely comparable to those of IMI/REL with AMX, with some variation in concentration of AMX across the subspecies. Concentrations of 1.5 µg/mL IMI, 0.75 µg/mL AVI and 8 µg/mL AMX were required to inhibit *M. abscessus* NCTC, whereas 16 µg/mL and 32 µg/mL AMX were required to inhibit growth of *M. abscessus* subsp. *bolletii* and *M. abscessus* subsp. *massiliense* respectively, at the same IMI/AVI concentrations (Fig. 7). Slightly lower concentrations of IMI and AVI (0.75 µg/mL and 0.375 µg/mL respectively) alongside 16 µg/mL AMX were needed to inhibit the growth of *M. abscessus* subsp. *abscessus* (Fig. 7). In the absence of AMX, IMI and AVI alone were able to slightly inhibit growth of *M. abscessus* subspecies at concentrations of 1.5 µg/mL IMI and 0.75 µg/mL AVI, however, not to the extent of inhibition seen with the addition of AMX (Fig. 7). Conversely, AMX (up to 32 µg/mL) was not able to inhibit the growth of *M. abscessus* alone (Fig. 7). The concentrations required to be bactericidal against *M. abscessus* subspecies were 1.5 µg/mL IMI, 0.75 AVI and 8 µg/mL AMX against *M. abscessus* NCTC and 1.5 µg/mL IMI, 0.75 AVI and 16 µg/mL AMX against *M. abscessus* subsp. *abscessus*. However, a slightly higher concentration of AMX (32 µg/mL) was required to sterilise *M. abscessus* subsp. *bolletii* and *M. abscessus* subsp. *massiliense* with the same concentrations of IMI (1.5 µg/mL) and AVI (0.75 µg/mL) (Table 1).

### 3.4 Imipenem combinations

**Fig. 8:**
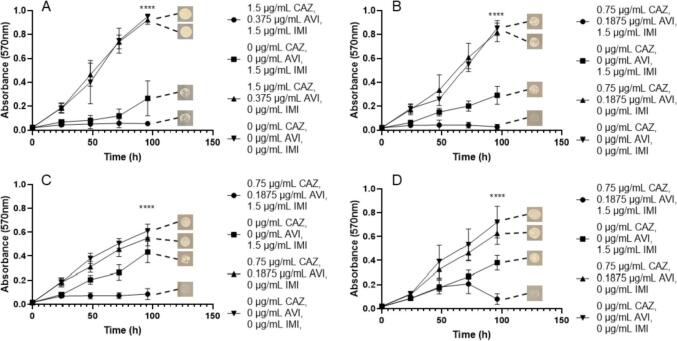
Growth curves of *M. abscessus* subspecies with ceftazidime (CAZ)/avibactam (AVI) vs imipenem (IMI). Endpoint solid media bacterial re-growth is shown at the end of each curve. A) *M. abscessus* NCTC 13031 with an MIC of 1.5 µg/mL CAZ, 0.375 µg/mL AVI and 1.5 µg/mL IMI (p ≤ 0.0001, n = 4). B) *M. abscessus* subsp. *abscessus* with an MIC of 0.75 µg/mL CAZ, 0.1875 µg/mL AVI and 1.5 µg/mL IMI (p ≤ 0.0001, n = 4). C) *M. abscessus* subsp. *bolletii* with an MIC of 0.75 µg/mL CAZ, 0.1875 µg/mL AVI and 1.5 µg/mL IMI (p ≤ 0.0001, n = 4). D) *M. abscessus* subsp. *massiliense* with an MIC of 0.75 µg/mL CAZ, 0.1875 µg/mL AVI and 1.5 µg/mL IMI (p ≤ 0.0001, n = 4).

Since IMI is a frontline antimicrobial used against *M. abscessus* infection, further investigation into the relationship between IMI and CAZ/AVI was necessitated. We assessed the efficacy against each *M. abscessus* subspecies and the MIC for *M. abscessus* subsp. *abscessus*, *M. abscessus* subsp. *bolletii* and *M. abscessus* subsp. *massiliense* was 0.75 µg/mL CAZ, 0.1875 µg/mL AVI and 1.5 µg/mL IMI (Fig. 8). A higher concentration of CAZ (1.5 µg/mL) and AVI (0.375 µg/mL) was needed to inhibit the growth of *M. abscessus* NCTC with the same concentration of IMI (1.5 µg/mL) as other subspecies (Fig. 8). For each subspecies, CAZ and AVI alone (up to 1.5 µg/mL and 0.375 µg/mL respectively) does not inhibit growth, however, IMI alone at 1.5 µg/mL does provide some growth inhibition, but less than the triplicate combination (Fig. 8). Higher concentrations of IMI are required for bactericidal activity against each subspecies, with MBCs of 0.75 µg/mL CAZ, 0.1875 µg/mL AVI and 3 µg/mL IMI. For *M. abscessus* subsp. *massiliense*, 6 µg/mL IMI is required alongside 0.75 µg/mL CAZ and 0.1875 µg/mL AVI in order to sterilise the bacteria (Table 1).

## 4 Discussion

The *M. abscessus* complex comprises of multiple subspecies of bacteria that are responsible for highly antimicrobial resistant infections, which are difficult to successfully treat. We have evaluated the β-lactamase inhibitors REL and AVI, both of which are highly effective agents which vastly increase the efficacy of β-lactams against each subspecies of the *M. abscessus* complex. We explored the optimal ratios of β-lactam partner agent to β-lactamase inhibitor against *M. abscessus*, in order to ascertain whether the commercially available formulations of IMI with REL and CAZ with AVI performed sub-optimally. We identified that REL was an effective β-lactamase inhibitor in conjunction with IMI down to a ratio of 2:1 (IMI:REL), which is the available pre-formulation (Fig. 1). The increase in REL concentration to IMI (1:1 and 1:2 (IMI:REL) ratios) was not sufficient to reduce the required inhibitory concentrations of IMI below 0.75 µg/mL, nor to reduce the bactericidal concentration below 3 µg/mL. Increasing the IMI:REL ratio to 4:1 allowed for the inhibitory concentration of IMI to remain at 0.75 µg/mL, but was not sufficient to sterilise the *M. abscessus* at 3 µg/mL of IMI. Therefore, the pre-formulated ratio of 2:1 (IMI:REL) is the most potent choice for activity against *M. abscessus*, as it provides the lowest dosing concentrations, whilst maintaining the most effective bactericidal activity of each assessed ratio. Conversely, we concluded that CAZ has no effect on the growth of *M. abscessus* at any ratio with AVI, therefore rendering the ratio used redundant in terms of treatment of *M. abscessus* infections.

Each triplicate combination that was assessed was capable of sterilising *M. abscessus* complex members down to low, therapeutically viable concentrations *in vitro* (Table 1). However, the most effective combination in terms of lowest combined MIC and MBC values was the grouping of IMI/AVI with MER, with concentrations of 0.375 µg/mL, 0.1875 µg/mL and 0.5 µg/mL respectively, for almost each subspecies (Fig. 4). This was closely followed by the combination of IMI/REL with MER, with combined concentrations of 0.75 µg/mL, 0.375 µg/mL and 1 µg/mL respectively for every *M. abscessus* subspecies (Fig. 2). The addition of CAZ/AVI to IMI also yields MIC values within a similar range, with combination concentrations of 0.75 µg/mL, 0.1875 µg/mL and 1.5 µg/mL respectively (Fig. 8). Conversely, the MBC values for this combination require a higher concentration of IMI (up to 6 µg/mL) for sterilisation of *M. abscessus* (Table 1). However, replacing IMI with 2 µg/mL MER in combination with CAZ/AVI results in bactericidal activity against all but one of the *M. abscessus* subspecies (Table 1). The concentrations of these combinations are much lower than the required inhibitory and bactericidal concentrations for IMI and MER both as independent agents, and also in combination with each other in the absence of a β-lactamase inhibitor (Table 1). Each combination of β-lactamase inhibitor increased the susceptibility of the *M. abscessus* complex to AMX, but at significantly higher concentrations of AMX (between 8 and 32 µg/mL) than when compared to either IMI or MER as an additional companion β-lactam (Table 1).

Each of these concentrations are achievable within the epithelial lining fluid (ELF), within the lungs where the majority of *M. abscessus* infections occur. Specifically, the maximum concentration of MER after multiple doses in the ELF is ∼7.07 µg/mL, whereas IMI is ∼9.76 µg/mL (Allegranzi et al., 2000, Rizk et al., 2018). Furthermore, the maximum concentration of AVI in the ELF during standard treatment is ∼5.1 µg/mL, which is comparable to REL at ∼5.33 µg/mL (Nicolau et al., 2015, Rizk et al., 2018). The concentration of CAZ that can be reached in ELF is ∼23.2 µg/mL, whereas AMX has a maximum ELF concentration of ∼0.89 µg/mL (Nicolau et al., 2015, Cook et al., 1994) It is therefore possible for each of these combinations to reach a clinically relevant concentration during treatment, since those combinations with higher AMX MIC/MBCs can be driven to within the range for ELF penetration with an increase to their respective β-lactam/β-lactamase inhibitor partners (Lopeman et al., 2020). As a frontline treatment drug for *M. abscessus* infection, it is plausible for combinations containing IMI to be implemented into the current treatment regime. However, since AVI is available only pre-formulated with CAZ (at a ratio of 4:1), rather than alone, any use of this β-lactamase inhibitor would include an excessive dosing of an unnecessary antibiotic which has numerous avoidable side-effects, the most extreme of which include seizures and severe skin reactions (Shirley 2018). Conversely, since REL is pre-formulated with IMI (and cilastatin), the additional antimicrobial burden on the patient is minimised, since IMI (with cilastatin) will be administered regardless within the frontline treatment regime for *M. abscessus* infections. The combination of IMI and REL has no additional side effects beyond those of IMI alone, so it is therefore a rational argument to utilise IMI and REL in place of IMI alone, alongside the implementation of MER, to limit the unnecessary drug burden of patients and successfully achieve bactericidal concentrations to sterilise *M. abscessus* infections. Therefore, we propose the use of IMI, REL and MER as the best option, from the combinations we have assessed, to potentially treat *M. abscessus* complex infections, because of the low MIC and MBC values yielded, as well as the best option for patient quality of life in terms of overall antimicrobial burden.

## 5 Conclusions

We have identified that the combinations of IMI, AVI and MER and IMI, REL and MER are the best triplicate combinations that we tested. These combinations yielded low MIC/MBC values of 0.375 µg/mL IMI, 0.1875 µg/mL AVI, 0.5 µg/mL MER and 0.75 µg/mL IMI, 0.375 µg/mL REL and 1 µg/mL MER. However, due to the fact that AVI is only available in a pre-formulation with CAZ (at a 4:1 ratio), there would be excessive additional antibiotic burden on patients that would not be the case with REL, since it is available pre-formulated with IMI. Therefore, the use of IMI, REL and MER against members of the *M. abscessus* complex is an appropriate choice based upon patient quality of life and sterilisation of *M. abscessus* infections.

### CRediT authorship contribution statement

**James Harrison:** Conceptualization, Formal analysis, Methodology. **John A. Weaver:** Conceptualization, Formal analysis, Methodology. **Maya Desai:** Funding acquisition, Methodology. **Jonathan A.G. Cox:** Conceptualization, Formal analysis, Funding acquisition, Methodology, Supervision.

## Declaration of Competing Interest

The authors declare that they have no known competing financial interests or personal relationships that could have appeared to influence the work reported in this paper.
